# Microplastics and the Kidney: A Narrative Review of Human Evidence, Molecular Mechanisms of Cell Injury, and Future Directions

**DOI:** 10.3390/ijms27208978

**Published:** 2026-10-10

**Authors:** Edoardo La Porta, Micaela Gentile, Marco Quaglia, Serena Ducoli, Sara Massardo, Daniela Verzola, Nicoletta Mancianti, Pasquale Esposito

**Affiliations:** 1 UOC Nephrology, IRCCS Istituto Giannina Gaslini, 16147 Genoa, Italy; 2 UOC Nephrology, Guglielmo da Saliceto Hospital, AUSL Piacenza, 29121 Piacenza, Italy; 3 Department of Translational Medicine, IRCCS AOU “SS Antonio e Biagio e Cesare Arrigo” University Hospital, 15121 Alessandria, Italy; 4 INSTM Unit of Brescia, Department of Mechanical and Industrial Engineering, University of Brescia, 25123 Brescia, Italy; 5 CNR-ICMATE, Institute of Condensed Matter Chemistry and Technologies for Energy, National Research Council, 16149 Genoa, Italy; 6 Department of Internal Medicine, University of Genoa, 16132 Genoa, Italy; 7 Nephrology, Dialysis and Transplantation Unit, Department of Emergency-Urgency and Transplantation, University Hospital of Siena, 53100 Siena, Italy; 8 Department of Internal Medicine and Medical Specialties (DIMI), University of Genoa, 16132 Genoa, Italy; 9 Nephrology, Dialysis and Transplantation Unit, AOM—IRCCS Ospedale Policlinico San Martino, 16132 Genoa, Italy

**Keywords:** microplastics, nanoplastics, kidney, nephrotoxicity, chronic kidney disease, dialysis, oxidative stress, biomonitoring

## Abstract

Microplastics (MPs) are widespread environmental contaminants, but their potential effects on kidney health remain poorly defined. As an organ continuously exposed to circulating particles and involved in their urinary elimination, the kidney may represent both a target of MP toxicity and a route of excretion. In this narrative review, we examine current evidence linking MPs to kidney injury, integrating experimental, mechanistic, and human data. Particular attention is given to the early events governing particle–cell interactions. After exposure, micro- and nanoplastics acquire an eco- and biomolecular corona that modifies their surface properties, cellular recognition, uptake, and biodistribution. Following internalization through phagocytic and endocytic pathways, particles may accumulate within endolysosomal compartments and interact with intracellular structures, disrupting cellular homeostasis. In renal cells, these events converge on mitochondrial dysfunction, reactive oxygen species generation, endoplasmic reticulum stress, inflammatory signaling, altered autophagy, and regulated cell death. Several interconnected pathways have been implicated, including Keap1–Nrf2/ARE and NF-κB signaling, MAPK and IRE1/XBP1-mediated stress responses, ACSL4–GPX4-related ferroptosis, and TGF-β1-related profibrotic signaling. Human studies have detected MPs in urine and kidney tissue and provide preliminary evidence linking MP burden with early markers of kidney damage, although current findings remain limited by small study populations and substantial methodological heterogeneity. Patients with chronic kidney disease, particularly those receiving dialysis, may represent a population of special interest because potentially altered renal handling may coexist with repeated exposure to polymer-based medical devices and dialysis fluids. Despite this potential relevance, data on MP exposure and accumulation in CKD and dialysis patients remain remarkably scarce. We finally discuss the limitations of current experimental models and analytical methods and the need for standardized biomonitoring, more environmentally relevant particles and exposure concentrations, and detection strategies capable of reaching the nanoscale, including artificial intelligence-assisted analysis. Overall, available evidence provides a mechanistic basis for MP-induced kidney injury, but its clinical relevance and contribution to human kidney disease remain to be established.

## 1. Introduction

Chronic kidney disease (CKD) represents a major global health challenge, affecting approximately 850 million individuals worldwide and projected to become one of the leading causes of death by 2050 [1], yet it remains largely under-recognized despite its growing clinical and societal impact [2]. In parallel with the global rise in chronic diseases, recent decades have also witnessed an unprecedented increase in environmental plastic pollution, progressively transforming microplastic exposure into a planetary-scale issue. Global plastic production increased from approximately 2 million tons in 1950 to nearly 475 million tons in 2022 and is projected to exceed 1.2 billion tons by 2060, while billions of tons of plastic waste currently contaminate terrestrial and aquatic ecosystems [3]. Less than 10% of plastic waste is effectively recycled. Environmental exposures are estimated to account for approximately 22% of the global disease burden according to the World Health Organization and are increasingly recognized as relevant, yet potentially modifiable, determinants of human health [4].

Within this evolving landscape of environmental risk factors, microplastics (MPs) have rapidly emerged as pervasive and biologically active contaminants. MPs are generally classified as primary MPs, intentionally manufactured at microscopic size for industrial applications, or secondary MPs, generated by fragmentation and degradation of larger plastic materials through physical, chemical, and biological processes, including ultraviolet radiation and microbial degradation. In the environment, MPs also act as substrates for microbial colonization, contributing to the formation of the so-called *plastisphere*, while simultaneously adsorbing and transporting other environmental pollutants, including per- and polyfluoroalkyl substances (PFAS), bisphenols, and heavy metals [5].

Microplastics (MPs) and nanoplastics (NPs) are increasingly recognized as ubiquitous environmental contaminants and potential human health hazards. Human exposure occurs predominantly through ingestion and inhalation, and MPs have been detected in several human biological fluids and tissues, including blood, placenta, lung, brain tissue and brain tumor, carotid atherosclerotic plaques, cerebrospinal fluid, urine, and kidney tissue [5,6,7,8,9,10].

Growing evidence further suggests potential reproductive and developmental effects, including associations with diminished ovarian reserve and possible prenatal and early-life effects such as placental transfer, endocrine disruption, and impaired fetal growth [11,12]. In addition, higher stool MP burden has been associated with colorectal cancer risk [13,14], supporting the potential involvement of MPs in human disease through inflammatory, oxidative, endocrine, and metabolic pathways.

Their detection in human biological matrices indicates that plastic particles can reach internal organs and supports the need to investigate their potential biological and clinical consequences. However, the extent to which MP exposure translates into clinically relevant organ injury remains incompletely understood. Recent literature has also attempted to integrate clinical and epidemiological observations into broader frameworks of possible MNP-related systemic effects; however, such proposed clinical constructs remain preliminary and require independent validation [15].

The kidney may be of particular interest because of its role in filtration and excretion and its continuous exposure to circulating substances. The presence of MPs in human urine and kidney tissue was first reported by Massardo et al. [7] using micro-Raman spectroscopy, with particles ranging from 1 to 29 μm identified in both matrices. Similar findings were subsequently reported by Pironti et al. [16], providing further evidence of renal exposure to MPs in humans. Experimental studies have additionally suggested that MPs and NPs may exert renal effects through oxidative stress, inflammation, mitochondrial dysfunction, podocyte injury, and tubular damage. Kidney organoid models have also reported impaired nephrogenesis following NPs exposure, while higher MPs concentrations have been detected in renal cell carcinoma tissue compared with adjacent healthy renal parenchyma [17]. These findings provide biological plausibility for potential renal effects but do not establish that MP exposure causes kidney dysfunction in humans.

Importantly, nephrology-specific clinical evidence remains extremely limited. Available human studies are few, generally based on small populations, and characterized by substantial methodological heterogeneity, including differences in sampling, particle characterization, and analytical techniques. Miguela-Benavides et al. [18] reported an association between urinary MP concentrations and urinary albumin-to-creatinine ratio (uACR) in 50 healthy volunteers. However, the cross-sectional design and small sample size preclude conclusions regarding temporality or causality, while the clinical significance of this association remains uncertain. Thus, current human evidence should be considered preliminary and hypothesis-generating rather than evidence of a causal relationship between MP exposure and kidney injury. This evidence gap is particularly relevant for patients with chronic kidney disease (CKD) and end-stage kidney disease (ESKD), who may represent clinically important populations for future investigation but remain insufficiently studied.

This narrative review is based on a literature search performed in PubMed, restricted to indexed peer-reviewed publications and focused on recent studies in the nephrological and urological fields. Search terms combined “microplastics” and “nanoplastics” with “kidney”, “renal”, “nephrotoxicity”, “chronic kidney disease” and “dialysis”, with priority given to articles published between 2020 and 2026.

We summarize the current evidence on MPs and kidney health, focusing on human and experimental data, potential mechanisms of renal toxicity, the relevance of MP exposure in patients with kidney disease, and the methodological limitations affecting interpretation of the available literature. We also identify key research priorities needed to determine whether MP exposure has clinically meaningful consequences for renal function and kidney disease progression.

Emerging approaches based on artificial intelligence (AI) and machine learning may further support this field by facilitating the automated detection and characterization of micro- and nanoplastics (MNPs), integrating complex exposure and clinical datasets, and potentially identifying patterns linking particle characteristics with renal outcomes. However, the application of these technologies to nephrology-focused MP research remains at an early stage and requires validation against standardized analytical and clinical frameworks.

## 2. In Vivo Evidence of Microplastic-Induced Renal Injury in Animal Models

Animal studies investigating the renal effects of MPs have predominantly relied on male C57BL/6 mice orally exposed to polystyrene (PS) microspheres through drinking water, with exposure durations ranging from a few weeks to six months and concentrations typically around 10 mg/L [19,20]. These concentrations greatly exceed human exposure levels and are best regarded as a means of compressing a chronic process into a tractable experimental window. This experimental paradigm reflects the broader trend in the literature, in which ingestion represents the main route of exposure and PS is by far the most extensively investigated polymer, whereas environmentally abundant polymers such as polyethylene (PE) and polypropylene (PP) remain comparatively underexplored.

Across these experimental models, MPs consistently induce dose- and size-dependent renal injury. Histopathological analyses have demonstrated vacuolar and granular degeneration of tubular epithelial cells, thickening of the glomerular basement membrane, mesangial matrix expansion, and, following prolonged exposure, overt tubulointerstitial fibrosis. Functional impairment has also been reported, with increased blood urea nitrogen (BUN) and serum creatinine representing the most consistent indicators of renal dysfunction [19]. More recently, nuclear imaging techniques, including ^99m^Tc-DMSA and ^99m^Tc-DTPA scintigraphy, have provided in vivo evidence of impaired renal uptake and filtration following exposure to polyethylene MPs, one of the most environmentally relevant yet comparatively under-investigated polymers.

Mechanistic studies have considerably expanded our understanding of MPs-induced nephrotoxicity. Using RNA sequencing, Xiong et al. [21] demonstrated that polystyrene particles spanning the nano- and microscale (80 nm, 0.5 μm, and 5 μm) activate distinct transcriptional programs. Nanoplastic particles (80 nm) predominantly induced immune and inflammatory pathways, whereas the larger microplastic particles mainly affected genes involved in circadian rhythm regulation, suggesting that particle size itself is an important determinant of the renal injury phenotype. These observations are consistent with in vitro studies in human proximal tubular epithelial (HK-2) cells, where PS-MPs induced oxidative stress, mitochondrial dysfunction, and apoptosis, supporting a direct cytotoxic effect on renal tubular cells [19].

Increasingly, experimental studies are moving beyond single-exposure models toward more realistic co-exposure paradigms. Zou et al. [22] demonstrated that MPs exacerbate cadmium-induced nephrotoxicity by increasing metal bioavailability and amplifying oxidative stress, autophagy, apoptosis, and fibrotic responses. Similar synergistic effects have been observed in avian models exposed to polyvinyl chloride (PVC) MPs and cadmium, where oxidative stress-mediated mitochondrial injury and apoptosis were accompanied by activation of the Nrf2/Keap1 antioxidant pathway [23]. Likewise, Pan et al. [20] identified accelerated tubular epithelial cell senescence as a novel mechanism through which PS-MPs promote renal fibrosis. At the tissue level, single-cell RNA sequencing has further shown that combined exposure to MPs and a high-fat diet profoundly remodels the renal microenvironment, promoting the expansion of pro-fibrotic PF4^+^ macrophage populations [24].

Recent evidence also suggests that kidney injury may develop through indirect systemic mechanisms. Zhang et al. [25] identified the gut–kidney axis as a key mediator of MPs-induced nephrotoxicity, demonstrating that intestinal exposure to MPs alters lipid metabolism through activation of the ACSL4/LPCAT3 pathway, ultimately inducing ferroptosis in renal tissue. These findings indicate that renal injury may occur even without substantial particle accumulation within the kidney itself, highlighting the systemic nature of MP toxicity. Complementing these observations, Meng et al. [26] demonstrated activation of necroptosis through the RIP1/RIP3/MLKL pathway in an avian model exposed to PS-MPs, expanding the spectrum of programmed cell death mechanisms involved beyond apoptosis.

Although experimental evidence supports the nephrotoxic potential of MPs, direct demonstration of particle deposition in human kidney tissue has only recently become available. Massardo et al. [7] provided one of the first direct demonstrations of MPs in both human urine and kidney tissue using micro-Raman spectroscopy. This finding establishes an important translational bridge between experimental observations and human disease, reinforcing the biological plausibility of MPs accumulation in renal tissue and its potential contribution to kidney injury.

**Figure 1:**
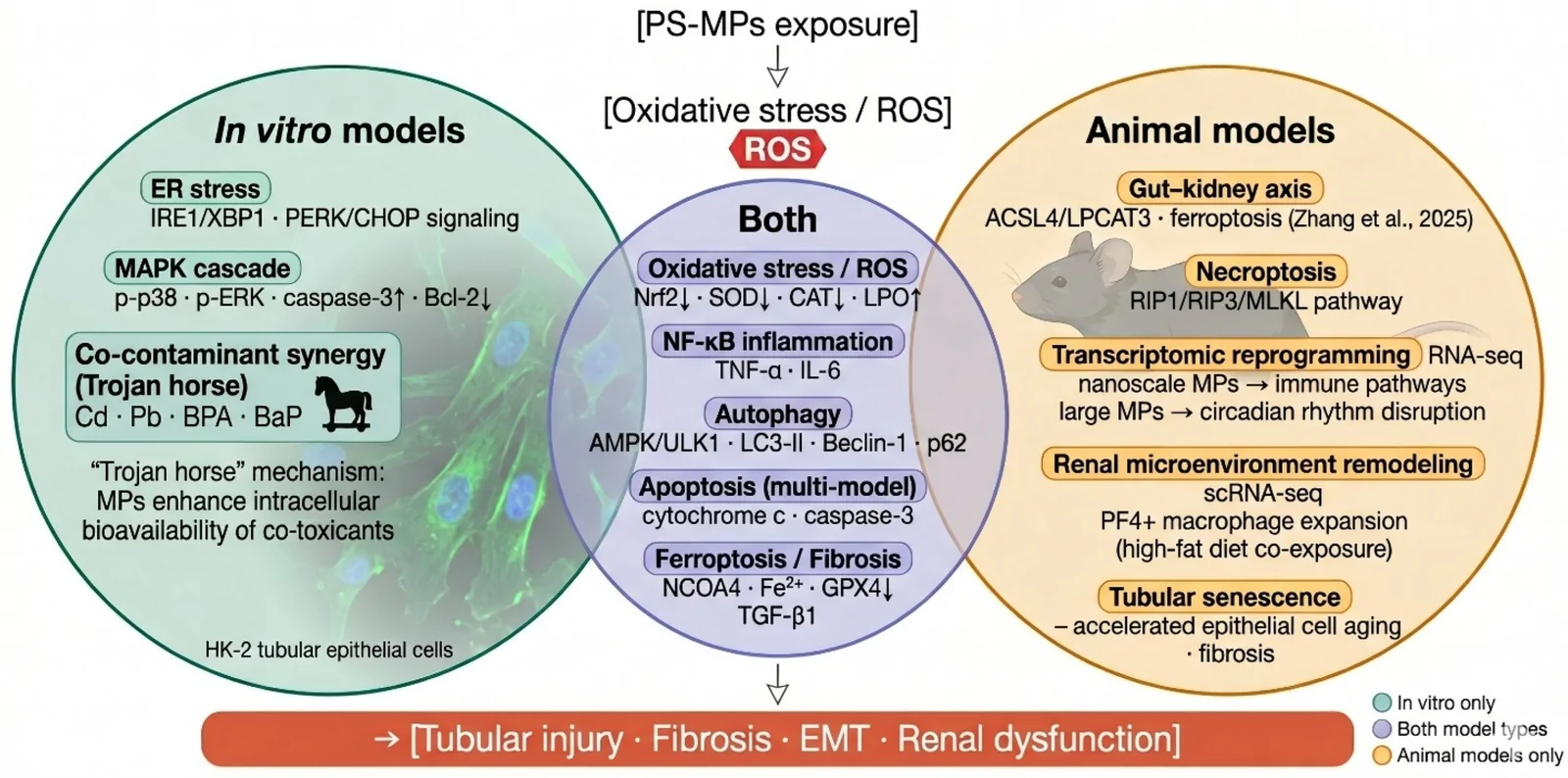
Mechanisms of polystyrene microplastic (PS-MP) induced renal injury: evidence from experimental models. The figure summarises evidence obtained almost exclusively with polystyrene particles, which account for the large majority of published mechanistic studies: evidence from experimental models. PS-MPs trigger ROS generation and oxidative stress in renal cells, activating interconnected pathways of ER stress, apoptosis, inflammation, autophagy, ferroptosis, necroptosis and senescence, which converge on tubular injury, glomerular damage, EMT and progressive renal dysfunction. Mechanisms are colour-coded by experimental model: in vitro (HK-2, HEK293; teal), animal models (C57BL/6 mice; amber), or both (purple). Supporting references: oxidative stress, ER stress, inflammation and autophagy [19,22,28,29,30]; tubular senescence and fibrosis [20]; size-dependent transcriptional responses [21]; renal microenvironment remodeling [24]; gut–kidney axis and ACSL4/LPCAT3-mediated ferroptosis (Zhang et al., 2025) [25]; necroptosis [26]. Abbreviations: ACSL4, acyl-CoA synthetase long-chain family member 4; AMPK, AMP-activated protein kinase; BaP, benzo[a]pyrene; Bcl-2, B-cell lymphoma 2; BPA, bisphenol A; CAT, catalase; Cd, cadmium; CHOP, C/EBP homologous protein; EMT, epithelial-to-mesenchymal transition; ER, endoplasmic reticulum; ERK, extracellular signal-regulated kinase; GPX4, glutathione peroxidase 4; HK-2, human kidney-2 proximal tubular epithelial cells; IL-6, interleukin-6; IRE1, inositol-requiring enzyme 1; LC3-II, microtubule-associated protein light chain 3-II; LPCAT3, lysophosphatidylcholine acyltransferase 3; LPO, lipid peroxidation; MAPK, mitogen-activated protein kinase; MLKL, mixed lineage kinase domain-like protein; MPs, microplastics; NCOA4, nuclear receptor coactivator 4; NF-κB, nuclear factor kappa-light-chain-enhancer of activated B cells; Nrf2, nuclear factor erythroid 2-related factor 2; p-, phosphorylated; p38, p38 mitogen-activated protein kinase; p62, sequestosome-1 (SQSTM1); Pb, lead; PERK, protein kinase R-like endoplasmic reticulum kinase; PF4, platelet factor 4; PS-MPs, polystyrene microplastics; RIP1/RIP3, receptor-interacting protein kinase 1/3; (sc)RNA-seq, (single-cell) RNA sequencing; ROS, reactive oxygen species; SOD, superoxide dismutase; TGF-β1, transforming growth factor beta 1; TNF-α, tumour necrosis factor alpha; ULK1, unc-51 like autophagy activating kinase 1; XBP1, X-box binding protein 1.

Overall, the integration of animal, cellular, and emerging human evidence consistently indicates that MPs may induce kidney injury through multiple complementary mechanisms, including direct cellular toxicity, oxidative stress, inflammation, mitochondrial dysfunction, metabolic dysregulation, ferroptosis, and interactions with environmental co-contaminants. Nevertheless, standardized experimental protocols together with well-designed longitudinal human studies remain essential to clarify the extent of renal accumulation, identify clinically relevant exposure thresholds, and define the true contribution of MPs to kidney disease [27]. The major experimental mechanisms underlying PS-MP-induced kidney injury are summarized in Figure 1. Mechanistically, the MPs uptake, lysosomal stress and ROS generation are expected to be largely polymer-independent, since driven by the particulate nature of the plastic debris rather than belonging to a specific polymer. Polymer type, surface charge, shape and associated additives are instead likely to modulate toxicity and the relative contribution of individual downstream pathways. Consistent with this, exposure to polyethylene MPs has been shown to impair renal uptake and filtration in vivo, although mechanistic characterisation for non-PS polymers remains limited.

## 3. Molecular Mechanisms of Micro- and Nanoplastic Cell Interactions: Corona Formation, Cellular Uptake, and Intracellular Fate

Although this review primarily focuses on microplastics (MPs), NPs are also considered in this section because particle size critically influences corona formation, cellular uptake, and intracellular trafficking. The term micro- and nanoplastics (MNPs) is therefore used throughout this section.

Micro- and nanoplastics (MNPs) interact with biomolecules both in the environment and after entering biological systems. In environmental matrices, adsorption of proteins, lipids, polysaccharides, microorganisms, and other organic compounds leads to the formation of an eco-corona. This process modifies the original physicochemical characteristics of the particles, including surface charge, hydrophobicity, aggregation state, and apparent size, and may consequently influence their stability, bioavailability, cellular interactions, and toxicity [31,32]. Therefore, environmentally exposed MNPs may have biological properties that differ considerably from those of pristine particles commonly used in experimental studies. After human exposure, MNPs further interact with biomolecules present in gastrointestinal fluids, plasma, and other biological matrices, resulting in the formation of a biomolecular or protein corona. Corona composition changes over time according to the biological environment and to the affinity of different molecules for the particle surface. Proteins with higher affinity tend to form a relatively stable inner layer, generally referred to as the hard corona, whereas more loosely associated and rapidly exchanging biomolecules constitute the soft corona. The resulting surface composition can influence how MNPs are recognized by cells and may modify their uptake, biodistribution, and biological effects [31,33].

The corona also appears to play a relevant role in cellular internalization. Experimental studies have shown that particles carrying an eco- or protein corona may be taken up differently from pristine particles, particularly by macrophages and other immune cells. Several mechanisms have been proposed, including phagocytosis, macropinocytosis, and receptor-mediated endocytosis, depending on particle size, surface properties, corona composition, and cell type. Corona-associated proteins may interact with Fc or scavenger receptors and facilitate cellular uptake, whereas other corona compositions may partially reduce particle recognition by immune cells [31,33]. Thus, the biological behavior of MNPs cannot be fully predicted from the characteristics of the original polymer alone.

For ingested MNPs, systemic distribution requires passage across the gastrointestinal barrier. Experimental evidence suggests that MNP exposure can affect the intestinal microbiota, alter epithelial tight junctions, and increase intestinal permeability, potentially facilitating particle translocation. Once particles reach the bloodstream, they may distribute to different tissues, including the kidney [32,34]. However, the mechanisms and efficiency of this process are still poorly defined and are likely to depend strongly on particle size, morphology, surface chemistry, and the acquired biomolecular corona.

Following cellular internalization, MNPs can enter endocytic compartments and lysosomes and may persist within the cytoplasm, where they interact with intracellular structures and interfere with cellular homeostasis. In renal cells, Wang et al. [19] provided direct evidence of concentration- and time-dependent internalization of polystyrene MPs in human proximal tubular epithelial HK-2 cells. Particle uptake was associated with mitochondrial superoxide generation, IRE1α-mediated endoplasmic reticulum stress, cPLA_2_/COX-1-related inflammatory signaling, MAPK activation, and inhibition of AKT/mTOR signaling, together with increased Beclin-1 and LC3-II expression and activation of autophagy (Figure 1). Verzola et al. [28] similarly observed time-dependent uptake in human proximal tubular cells, with particles mainly localized in the cytoplasm and in discrete clusters in the perinuclear region, without detectable nuclear localization.

These findings indicate that surface modification, corona formation, cellular uptake, and intracellular trafficking are closely related steps in determining the biological effects of MNPs. However, the mechanisms regulating these processes in human renal cells remain largely unknown, particularly for environmentally aged particles, heterogeneous particle mixtures, and polymers other than polystyrene.

## 4. Human Evidence and In Vitro Mechanisms of Renal Injury

Compared with the large body of experimental evidence, human studies investigating MPs and kidney health remain limited but are rapidly increasing. To date, MPs have been detected in both urine and kidney tissue, supporting the biological plausibility of renal exposure. The first direct demonstration of MPs in human urine and renal tissue was provided by Massardo et al. [7] using micro-Raman spectroscopy, identifying particles of different polymer compositions and sizes in both biological matrices. Similar findings were subsequently reported by Pironti et al. [16], further supporting the presence of MPs within the human urinary system, even if the number of samples analyzed (six) and the number of blanks (two) turn out to be underpowered.

Evidence regarding the actual accumulation of MPs in kidney tissue, however, remains inconsistent. Zhang et al. [17] identified MPs in peritumoral renal tissue with LDIR (matching degree > 0.65) and Py-GC/MS and reported higher concentrations in renal cell carcinoma specimens than in adjacent healthy parenchyma (total MPs, PE and PVC, respectively, *p* = 0.001, *p* = 0.002 and *p* < 0.001), suggesting preferential accumulation within pathological tissues. In contrast, Horvatits et al. [35] failed to detect MPs in kidney samples, highlighting the considerable variability among currently available studies. These discrepancies are likely attributable to differences in sampling procedures, contamination control, analytical techniques, and particle detection thresholds. Specifically, the available studies differ at every step of the analytical chain (Table 1). Tissue digestion ranged from alkaline protocols 10% KOH in Massardo et al. and in Pironti et al. [16] and a two-step KOH/NaOCl digestion followed by H_2_O_2_ and acetone defatting in Horvatits et al. to concentrated (68%) nitric acid in Zhang et al. Analytical approaches were equally heterogeneous: micro-Raman spectroscopy with visual inspection of the whole filter in Massardo et al. (spatial resolution ~1 µm; spectra matched against Renishaw, SLoPP and IRUG libraries by HQI and normalised correlation) and in Pironti et al. (HQI > 80); Nile red staining with fluorescence counting and Raman confirmation limited to 2–6 particles per sample (>70% match), in Horvatits et al. [35]; and a combination of LDIR (match > 0.65, performed on only two paired samples), SEM and mass-based Py-GC/MS restricted to 11 target polymers in Zhang et al. Contamination control also differed in rigour: Massardo et al. processed twelve procedural blanks, and additionally screened KOH pellets, deionised water, the KOH solution and pristine filters; Horvatits et al. processed seven procedural blanks, derived a formal LOD (mean + 3 SD, ≈3 particles/g) and blank-corrected all results, a threshold below which all three kidney samples fell; Zhang et al. processed three field blanks without reported blank subtraction; and Pironti et al. used one environmental and one procedural blank. Against this background, the apparently negative kidney result of Horvatits et al. should be read as concentrations below a relatively high detection limit rather than as evidence of absence; conversely, the concentration reported by Zhang et al. refers to a larger size class and to peritumoral tissue, and it should be noted that the “healthy” renal tissue analysed both by Massardo et al. and by Zhang et al. derives from tumour nephrectomies.

Emphasizing the urgent need for standardized methodologies.

Further evidence of renal exposure comes from biomonitoring studies. Nihart et al. [9] quantified MPs concentrations across several human organs and found that renal concentrations, predominantly polyethylene particles, were substantially lower than those observed in the brain but comparable to those measured in the liver. Although the kidney does not appear to be a major site of MPs accumulation, these findings indicate that it is continuously exposed to circulating particles and may therefore be susceptible to chronic toxic effects.

Clinical evidence linking microplastic (MP) exposure to kidney injury remains extremely limited. One of the first studies to investigate this association was conducted by Miguela-Benavides et al. [18], who analyzed urine and stool samples from 50 healthy volunteers using high-performance liquid chromatography coupled with high-resolution mass spectrometry (HPLC-HRMS). Six different polymers were detected in nearly half of the urine samples, and urinary MP concentrations were independently associated with the urinary albumin-to-creatinine ratio (uACR) (OR 2.18, 95% CI 1.17–4.01). Although these findings suggest an association between urinary MP exposure and a marker of renal function, their clinical significance remains uncertain. Importantly, the study had a small sample size and a cross-sectional design, which precludes establishing a temporal or causal relationship between MP exposure and kidney injury. Therefore, the observed association should be interpreted cautiously and requires confirmation in larger, longitudinal studies incorporating established clinical measures of kidney function and kidney injury.

Overall, available human evidence (Table 2) indicates that MPs can be detected in human biological fluids and, in some studies, within renal tissue, although the extent of renal accumulation remains uncertain. Current evidence is limited by small sample sizes, heterogeneous analytical methodologies, and the lack of standardized protocols. Larger, well-designed clinical studies are therefore required to clarify the relationship between MP exposure, tissue accumulation, and kidney disease.

The biological plausibility of these clinical observations is supported by an expanding body of in vitro evidence (Figure 1). Most mechanistic studies have employed human proximal tubular epithelial (HK-2) cells or embryonic kidney cells (HEK293), mainly exposed to polystyrene MNPs. Although experimental conditions vary considerably across studies, spanning human tubular cell lines, iPSC-derived kidney organoids, and rodent and avian in vivo models, as detailed in Table 2 and Table 3, oxidative stress and mitochondrial dysfunction emerge as central events linking particle exposure to inflammation, altered autophagy, different forms of regulated cell death, and ultimately fibrotic responses. The origin of each mechanistic observation should therefore be taken into account when extrapolating these findings to the human kidney [19,26,31].

Oxidative stress is the best-characterized mechanism of plastic particle-induced nephrotoxicity. Increased production of reactive oxygen species (ROS), particularly mitochondrial ROS, is accompanied by disruption of cellular antioxidant defenses, including Nrf2-related signaling, superoxide dismutase (SOD), and catalase (CAT), and by increased lipid peroxidation. ROS accumulation also promotes activation of NF-κB and the release of pro-inflammatory mediators, including TNF-α and IL-6. Mitochondrial dysfunction appears closely linked to this response, with alterations in mitochondrial membrane potential, increased mitochondrial ROS production, and activation of downstream stress and cell-death pathways. These effects appear to depend on particle concentration, size, and duration of exposure [19,26,29].

Several intracellular pathways connect oxidative and mitochondrial stress with cell injury. Activation of MAPK signaling, including p38 and ERK phosphorylation, has been associated with cytochrome c release, increased caspase-3 activity, reduced Bcl-2 expression, and apoptosis in renal tubular cells [29]. Endoplasmic reticulum (ER) stress represents another relevant mechanism. Activation of the IRE1/XBP1 and PERK/CHOP pathways links accumulation of misfolded proteins and oxidative stress to inflammatory signaling and apoptosis [19,30]. These pathways are not independent, and the available experimental evidence suggests substantial crosstalk between mitochondrial dysfunction, ER stress, and ROS generation.

Autophagy may represent an additional response to MNP-induced cellular stress. Changes in LC3-II, Beclin-1, and p62 expression have been reported in renal cell models, together with modulation of AMPK/ULK1 and AKT/mTOR signaling [19,22]. The biological significance of this response probably depends on the intensity and duration of exposure: activation of autophagy may initially contribute to the removal of damaged cellular components, whereas persistent or dysregulated autophagic signaling may accompany progressive cellular injury.

More recent studies have expanded the spectrum of regulated cell-death mechanisms implicated in MNP nephrotoxicity. Ferroptosis is characterized by iron-dependent lipid peroxidation and impairment of antioxidant defenses, particularly GPX4, and markers consistent with this pathway have been reported in renal tissue following MP exposure. The evidence derives, however, from the gut–kidney axis model discussed in Section 2 [25]. Direct validation of this mechanism in human renal cells is currently lacking, and the extent to which this mechanism operates as a specific renal pathway rather than as a consequence of systemic metabolic alterations remains to be established. In animal models, activation of the ACSL4/LPCAT3 pathway through the gut–kidney axis provides additional evidence linking altered lipid metabolism to renal ferroptosis [25].

Necroptotic pathways have also been described in an avian kidney model, including NF-κB-associated inflammatory signaling and RIP1/RIP3/MLKL-dependent necroptosis [26] although this pathway has not yet been validated in human renal cells.

These mechanisms may coexist rather than act as mutually exclusive forms of cell death.

With prolonged exposure, persistent oxidative stress, inflammation, mitochondrial dysfunction, and regulated cell death may shift the renal response towards maladaptive repair and fibrosis. Experimental studies in rodent models have reported tubular epithelial senescence, epithelial-to-mesenchymal transition, activation of TGF-β1-related signaling, and expansion of pro-fibrotic macrophage populations [20,24]. These findings suggest that the biological effects of MPs may extend beyond acute cytotoxicity and contribute to chronic remodeling of the renal microenvironment.

Beyond exposure to plastic particles alone, co-exposure models may better reflect real-world conditions, where MPs coexist with environmental contaminants and plastic-associated chemicals. MPs can adsorb other compounds on their surface and potentially modify their bioavailability and cellular delivery, a phenomenon commonly described as the “Trojan horse” effect. Experimental studies have reported enhanced toxicity following combined exposure to MPs and contaminants such as cadmium and bisphenol A. In human proximal tubular cells, Verzola et al. [28] showed that co-exposure to MPs and bisphenol A exacerbated cellular injury compared with either exposure alone. Similarly, in animal models, MPs enhanced cadmium-induced oxidative stress, mitochondrial dysfunction, autophagy, apoptosis, and fibrotic responses [22]. These observations suggest that the nephrotoxic effects of MPs should also be considered in the context of complex environmental exposures rather than as isolated particle toxicity.

More physiologically relevant experimental systems are also beginning to provide additional information on MNP-induced kidney injury. Human iPSC-derived kidney organoids exposed to polystyrene particles showed particle adhesion to glomerulus-like structures, reduced organoid growth after both short- and long-term exposure, and activation of ROS-dependent apoptotic pathways during nephrogenesis [36]. Although kidney organoids cannot fully reproduce the complexity of the mature human kidney, these findings provide evidence that plastic particle exposure may interfere with renal development and support their use as an intermediate experimental model between conventional cell cultures and in vivo studies.

### An Integrated Model of Microplastic-Induced Renal Injury

Taken together, the available evidence is more consistent with a self-amplifying sequence than with parallel pathways. Following the internalisation and lysosomal trafficking described in Section 3, particle persistence within the endolysosomal compartment compromises mitochondrial integrity, and the resulting increase in mitochondrial ROS exceeds the antioxidant capacity of Nrf2. Oxidative modification of the endoplasmic reticulum in turn triggers the unfolded protein response, which, through PERK/CHOP and IRE1/XBP1 signalling, further increases ROS production and establishes a feedback loop between the two mechanisms. Further, MAPK and NF-κB activation appears to diverge towards distinct outcomes: apoptosis where mitochondrial permeabilisation predominates, necroptosis where inflammatory signalling prevails, and ferroptosis where lipid peroxidation outpaces GPX4-dependent repair. Autophagy mechanism is positioned at this same level as a modifier rather than an effector, being cytoprotective at low exposure and contributing to injury once lysosomal capacity is saturated. Where exposure is sustained, or amplified by co-contaminants acting through the Trojan-horse effect, the repeated activation of these pathways shifts the tubular epithelium from repair towards the senescent and pro-fibrotic phenotype described above. This sequence, summarised in Figure 1, should be regarded as a signal pathway hypothesis to explain the current results rather than as an experimentally validated pathway: no single study has tested the model in its entirety, or aimed to link the different pathways to give an univocal interpretation and several of the proposed causal links are inferred from non-renal systems.

**Table 3:** The currently available human evidence on MPs detected in urine and kidney tissue.

Study (Year)	Sample	Method	Main Findings
Pironti et al. (2023) [16]	Urine	Raman spectroscopy	First evidence of MPs in human urine
Horvatits et al. (2022) [35]	Kidney tissue	FTIR-based analysis	MPs not detected in kidney samples (negative result not informative below the ~10–20 µm detection limit)
Massardo et al. (2024) [7]	Kidney tissue + urine	μRaman spectroscopy	First evidence of MPs in human kidney. MPs detected in both matrices (1–29 μm)
Song et al. (2024) [37]	Urine	Py-GC/MS and LDIR	Higher urinary MPs in urban populations
Rotchell et al. (2024) [38]	Urine	μFTIR spectroscopy	High variability among samples
Nihart et al. (2025) [9]	Multiple tissues including kidney	Quantitative analysis	Detectable kidney MPs burden
Zhang et al. (2025) [17]	Renal cancer tissue	Multimodal detection	Higher MPs burden in tumor tissue
Jahedi et al. (2025) [39]	Urine + respiratory samples	μFTIR spectroscopy	Lower urinary MPs compared with respiratory fluids
Dong et al. (2025) [40]	Urine	Optical microscopy/SEM	Association with behavioral outcomes
Miguela-Benavides et al. (2026) [18]	Urine	HPLC-HRMS	Urinary MPs associated with higher uACR
Liu et al. (2026) [41]	Urine	LDIR	Dietary habits associated with MPs burden

Approximate 1. µm; FTIR ~10–20 µm; µRaman ~1 µm; LDIR ~20 µm; Py-GC/MS no size cut-off (mass-based); optical microscopy/SEM n/a—no chemical identification. ***Abbreviations:*** MPs, microplastics; μFTIR, micro-Fourier transform infrared spectroscopy; μRaman, micro-Raman spectroscopy; LDIR, laser direct infrared spectroscopy; Py-GC/MS, pyrolysis–gas chromatography/mass spectrometry; HPLC-HRMS, high-performance liquid chromatography–high-resolution mass spectrometry; uACR, urinary albumin-to-creatinine ratio.

## 5. ESKD as a High-Risk Condition for Microplastic Exposure and Accumulation

Patients with end-stage kidney disease (ESKD) undergoing dialysis may represent a population of particular interest for MP exposure and accumulation. In addition to the possibility of altered renal handling of plastic particles, these patients experience chronic exposure to large volumes of treated water and prolonged contact between blood and polymer-based extracorporeal devices. Conventional hemodialysis (HD) exposes patients to approximately 300–600 L of treated water each week, while online hemodiafiltration (HDF) further increases exposure through convective transport of dialysis fluid [42].

Preliminary clinical evidence has explored whether dialysis patients may carry a greater systemic MP burden. In a small case–control study, Fiore et al. [42] reported higher circulating MPs concentrations in HD patients than in non-dialysis controls (9132 [4896–15,847] vs. 3554 [1661–7340] particles/g), although the difference narrowly missed statistical significance (*p* = 0.06), most likely because of the limited sample size.

Potential sources of dialysis-related MP exposure were comprehensively investigated by Passos et al. [43], who examined the entire dialysis water pathway and identified several possible contamination points, including reverse osmosis systems, storage tanks, distribution circuits, dialyzers, and PVC tubing. Based on previously reported MPs concentrations in drinking water, the estimated exposure ranged from 0.0021 to 3768 particles per week despite reverse osmosis treatment, with substantially higher theoretical values under filtration failure conditions. Importantly, many components of dialysis systems are themselves manufactured from synthetic polymers—including polysulfone, polyethersulfone, polyamide, polycarbonate, and polyvinyl chloride (PVC)—raising the possibility that long-term exposure may derive not only from incoming water but also from the dialysis equipment itself.

These findings also highlight an important limitation of current dialysis water quality standards. Existing regulations primarily focus on microbiological contamination and endotoxins, whereas particulate contaminants, including MPs and nanoplastics, are not routinely monitored. Furthermore, reverse osmosis systems were not specifically designed to remove plastic particles, particularly at the nanoscale. Membrane aging, oxidative degradation, filter fouling, and post-filtration plastic piping may therefore represent additional sources of contamination during long-term clinical use. Although the absolute particle concentrations appear low, repeated exposure over years of maintenance dialysis could result in substantial cumulative exposure.

Direct evidence that MPs are present in dialysis fluids has recently been provided by Kara et al. [44]. Using Fourier-transform infrared (FT-IR) spectroscopy, the authors detected MPs in all analyzed HD and peritoneal dialysis (PD) fluid samples. Fiber-shaped particles accounted for more than 60% of all detected MPs, while polyethylene (PE), PVC, and ethylene-vinyl acetate (EVA) were the most frequently identified polymers. Although MP concentrations were comparable between HD and PD fluids, particles recovered from HD samples were significantly larger. Because PD is performed continuously, cumulative weekly exposure was estimated to be greater in PD patients. More recently, Deng et al. [45] investigated the impact of an HD session on MP exposure by quantifying particles in blood samples collected before and after treatment using LDIR (match degree ≥ 0.80). Compared with pre-HD samples, the number of MPs was significantly higher after HD. However, these results should be interpreted cautiously because of the small study population (nine patients) and the absence of reported blank controls. Importantly, LDIR cannot identify particles below approximately 20 µm; thus, the detected post-HD increase is more consistent with particle release from the extracorporeal circuit than with backfiltration across the dialysis membrane. Direct evidence of MNP backfiltration into the bloodstream during haemodialysis is currently lacking, largely because of methodological limitations. To date, no study has directly compared MNP passage across high-flux versus low-flux dialysis membranes or between HD and haemodiafiltration (HDF).

Taken together, these findings indicate that dialysis patients may be exposed to MPs not only through conventional environmental pathways but also through dialysis-related medical devices and treatment fluids. This scenario is particularly relevant because dialysis membranes place circulating blood in prolonged contact with large volumes of treated fluid and synthetic polymeric materials. In high-flux HD and HDF, where convective transport enhances fluid exchange across the membrane, there is also the theoretical possibility that particles or plastic-associated additives could bypass the gastrointestinal and respiratory barriers that normally represent the primary routes of environmental exposure. More broadly, iatrogenic exposure through polymer-based medical devices and infusion fluids may represent an additional source of circulating MPs. In a multicountry analysis of intravenous infusion products, Casella et al. detected MPs in nearly all tested samples, with substantially higher particle concentrations in plastic bags than in glass containers, further supporting the relevance of medical-device-related exposure pathways [46].

This issue may be especially important in ESKD, a condition already characterized by chronic inflammation, oxidative stress, endothelial dysfunction, immune dysregulation, and accelerated vascular aging. Experimental evidence indicates that MPs, nanoplastics, and plastic-associated additives can induce reactive oxygen species generation, mitochondrial dysfunction, inflammatory activation, apoptosis, endocrine disruption, and microvascular injury [7]. Even if dialysis-related exposure contributes only modestly to the total MP burden, these biological effects could be amplified in patients with advanced kidney disease in the setting of potentially altered particle handling and their pre-existing pro-inflammatory milieu.

**Figure 2:**
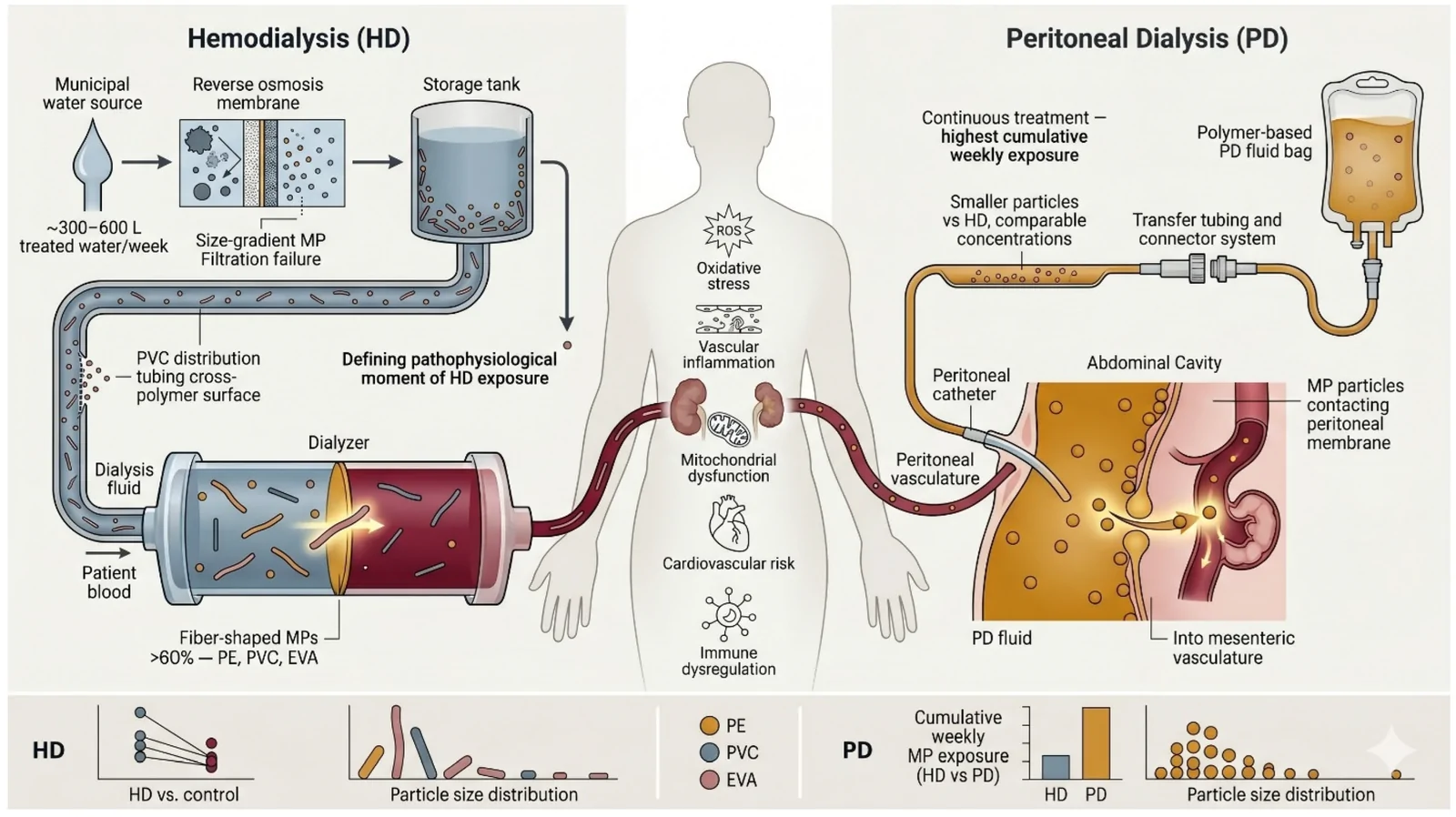
Microplastic exposure routes in end-stage kidney disease patients undergoing hemodialysis and peritoneal dialysis. Hemodialysis (**left**): incomplete size-dependent retention by reverse osmosis, storage tanks and PVC distribution tubing act as sequential contamination points along the ~300–600 L of treated water delivered weekly, while polysulfone-, polyethersulfone- and PVC-based circuit components represent an additional intrinsic particle source; fibre-shaped particles predominate (>60%; PE, PVC, EVA). Peritoneal dialysis (**right**): polymer-based fluid bags, transfer sets and the peritoneal catheter place MPs in continuous contact with the peritoneal membrane and mesenteric vasculature, yielding the highest estimated cumulative weekly exposure despite MP concentrations comparable to HD and a smaller particle size. Both modalities converge on oxidative stress, mitochondrial dysfunction, vascular inflammation, immune dysregulation and increased cardiovascular risk (centre). Lower panels summarise reported circulating MP burden, particle size distribution and estimated cumulative exposure by modality. **Abbreviations**: EVA, ethylene-vinyl acetate; HD, hemodialysis; MPs, microplastics; PD, peritoneal dialysis; PE, polyethylene; PVC, polyvinyl chloride.

Despite these observations, the clinical implications remain almost completely unknown. Several key questions are still unanswered, including the true concentrations of MPs and nanoplastics in dialysis water, dialysis fluids, and patients’ blood; the ability of different dialysis membranes to retain or remove particles according to their size, morphology, and polymer composition; the contribution of residual kidney function to MP clearance; and the long-term biodistribution and tissue accumulation of MPs in patients receiving chronic dialysis. Addressing these knowledge gaps will require standardized analytical methodologies together with dedicated translational and clinical studies specifically designed for the dialysis setting. The main potential routes of microplastic exposure in patients with ESKD undergoing hemodialysis and peritoneal dialysis are summarized in Figure 2.

## 6. Urinary Microplastics as a Non-Invasive Biomarker of Human Exposure

Beyond demonstrating that the kidney represents a target organ for MPs deposition and injury, the detection of MPs in urine has attracted growing interest as a non-invasive biomarker of human exposure. Because urine can be collected easily and repeatedly, urinary MPs may provide valuable information on systemic bioavailability, environmental exposure, and potentially on the renal handling and elimination of plastic particles. Consequently, increasing efforts have been devoted to evaluating urinary MPs as a biomonitoring tool and to identifying the environmental and behavioral factors influencing their presence.

Several studies have investigated the determinants of urinary MPs concentrations. Song et al. [37] analyzed urine samples using pyrolysis-gas chromatography/mass spectrometry (Py-GC/MS) and laser direct infrared spectroscopy (LDIR), demonstrating significantly higher MPs concentrations in individuals living in urban areas than in rural settings. The authors also identified the use of plastic toys and personal care products as potential behavioral risk factors for increased MP exposure.

Dietary habits have likewise emerged as important determinants of urinary MPs burden. Liu et al. [41] evaluated more than 1300 children, representing one of the largest epidemiological studies currently available in this field. Using LDIR, the authors reported a median urinary concentration of approximately 250 particles/mL, generally higher than those described in adult populations. Greater behavioral exposure during childhood—including frequent hand-to-mouth activity and extensive use of plastic toys—may also contribute to the increased urinary MP burden observed in pediatric populations [47].

This observation is likely explained, at least in part, by the lower spectral similarity threshold adopted for particle identification (65%) compared with the more stringent thresholds (75–80%) commonly applied in other studies. Lower thresholds are expected to increase false-positive polymer assignments. The reported values should therefore be considered indicative, and these results have to be interpreted with caution.

Urinary MPs have also been investigated in specific clinical conditions. Rotchell et al. [38] analyzed urine samples from women with and without endometriosis using micro-Fourier transform infrared (μFTIR) spectroscopy. Although no significant differences were observed between the two groups, MP concentrations showed remarkable interindividual variability, ranging from undetectable levels in some samples to more than 36,000 particles/L, highlighting the substantial heterogeneity that currently characterizes urinary MP measurements.

Similarly, Jahedie et al. [39] compared MPs concentrations in urine, sputum, and bronchoalveolar lavage fluid from patients with respiratory diseases using μFTIR spectroscopy. As expected, urinary MP concentrations were considerably lower than those measured in respiratory samples, consistent with the respiratory tract representing a primary route of environmental exposure. Interestingly, the polymer composition also differed among biological matrices, suggesting that urinary MPs likely reflect only the fraction of particles that successfully cross physiological barriers and become systemically bioavailable before renal excretion.

Beyond environmental exposure assessment, urinary MPs have also been explored as potential biomarkers of disease susceptibility. Dong et al. [40] investigated more than 1000 children aged 6–9 years and reported positive associations between urinary MPs concentrations and emotional problems, conduct disorders, hyperactivity, and impaired peer relationships, together with an inverse association with prosocial behavior. However, these findings should be interpreted with caution because particle identification relied primarily on optical microscopy and scanning electron microscopy, without spectroscopic confirmation of polymer composition. This methodological limitation increases the risk of particle misclassification and may have influenced the reported associations. The reported associations require replication using spectroscopic or thermoanalytical methods before they can be considered robust.

Growing evidence that MPs are able to cross physiological barriers, accumulate in distant organs, including the brain, and subsequently appear in urine has stimulated increasing interest in understanding their biodistribution, tissue relocation, and elimination kinetics in vivo [34]. In this context, urinary MPs may represent an accessible surrogate marker not only of environmental exposure but also of systemic particle bioavailability.

Overall, urine represents a unique and non-invasive matrix for human MPs biomonitoring, integrating information on environmental exposure, lifestyle-related risk factors, and systemic distribution. However, substantial heterogeneity in analytical techniques, sample preparation protocols, contamination control procedures, particle size detection limits, and polymer identification criteria currently limits comparisons across studies. The harmonization of analytical methodologies and the implementation of large-scale epidemiological investigations will therefore be essential to establish urinary MPs as reliable biomarkers of exposure and to clarify their relationship with kidney health.

## 7. Methodological Challenges and Future Perspectives in Microplastics Research

The rapid expansion of MPs research has been accompanied by remarkable methodological heterogeneity. Although this growing interest has substantially increased the number of published studies, it has also involved research groups with limited expertise in MP analysis, potentially affecting data quality and reproducibility. Because MPs are ubiquitous environmental contaminants, rigorous quality assurance and quality control (QA/QC) procedures—including procedural blanks, contamination monitoring, and standardized laboratory practices—are essential to minimize false-positive findings. Nevertheless, methodological variability remains one of the major obstacles preventing reliable comparisons across studies and the translation of current evidence into clinically meaningful conclusions. A largely underappreciated theme underlies most of these limitations: the particle size range that is biologically most relevant lies below the range that current analytical techniques can detect in biological matrices.

### 7.1. Methodological Challenges in Human Biomonitoring

One of the principal limitations of current human studies is the lack of standardized analytical workflows. Every step of the analytical process—including sample collection, storage, digestion, contamination control, particle isolation, and polymer identification—may influence the final results, making comparisons across laboratories extremely difficult. Biological matrices pose additional challenges because proteins, lipids, carbohydrates, and other endogenous components can interfere with particle isolation and identification. Moreover, mechanical abrasion and ageing processes, such as photochemical degradation and hydrolysis, can modify the physicochemical properties of MPs, leading to spectral peak shifts, signal interference, and obscuration of polymer-specific spectral signatures [48].

Different digestion protocols have been proposed, including alkaline (KOH, NaOH), oxidative (H_2_O_2_), acidic, and enzymatic approaches, each characterized by specific advantages and limitations depending on the biological matrix under investigation. Likewise, a broad range of analytical techniques—including micro-Fourier transform infrared spectroscopy (μFTIR), micro-Raman spectroscopy (μRaman), laser direct infrared spectroscopy (LDIR), pyrolysis-gas chromatography/mass spectrometry (Py-GC/MS), and high-performance liquid chromatography (HPLC)-based methods—are currently employed for MP detection. However, differences in analytical sensitivity, particle-size detection limits, and polymer-identification criteria substantially contribute to the heterogeneity observed across published studies [49]. Reported MP concentrations are not directly comparable across studies because analytical techniques differ not only in sensitivity but also in the range of particle sizes they can detect (µFTIR, ~10–20 µm; LDIR, ~20–50 µm; µRaman, ~1 µm). Since the size distribution of MPs in kidney and urological samples is likely skewed toward smaller particles, methods with a lower size-detection threshold may systematically yield higher particle counts even when applied to the same sample, thereby introducing methodological bias into cross-study comparisons. Py-GC/MS is not constrained by a particle-size detection threshold in the same way, but its results are generally expressed as polymer mass rather than particle number and therefore cannot be directly compared with particle-counting methods. Together with heterogeneity in spectral-matching thresholds, these methodological differences make accurate assessment of data reliability challenging; meaningful quantitative comparisons are most robust when studies use comparable analytical workflows, detection thresholds, and reporting metrics.

Among available techniques, micro-Raman spectroscopy offers particularly high spatial resolution and excellent adaptability to biological tissues and fluids. However, fluorescence interference, overlapping spectral bands, and operator-dependent spectral interpretation remain important limitations, especially when analyzing complex biological specimens. These issues may complicate polymer identification and increase the risk of misclassification, particularly for highly degraded particles or particles embedded within an organic matrix. In addition, differences in spectral libraries, preprocessing algorithms, and criteria used for spectral matching may further reduce reproducibility across laboratories and should be considered when comparing results from different studies. Fluorescence is one of the most significant challenges in Raman spectroscopy: the fluorescence background, particularly in labeled or environmentally weathered MPs, can overwhelm the relatively weak Raman signal in complex biological matrices and mask the vibrational bands required for polymer identification and discrimination [50].

To address these challenges, SpeComp, a freely available open-source software package, was recently developed to improve Raman-based identification of MPs in biological matrices [51]. SpeComp incorporates standardized preprocessing procedures, including baseline correction, denoising, normalization, and substrate-signal subtraction, while comparing unknown spectra with curated reference libraries through multiple similarity algorithms. Automated similarity scoring reduces operator-dependent variability, improves reproducibility, and facilitates discrimination between polymer spectra and biological background signals. In our recent study reporting the first detection of MPs in human urine and kidney tissue, SpeComp substantially improved spectral interpretation despite fluorescence interference associated with 633 nm Raman acquisition, illustrating the potential of computational approaches to enhance analytical robustness.

Artificial intelligence is expected to further improve MP characterization. Machine-learning algorithms can enhance spectral preprocessing, automate particle recognition, improve Raman mapping, and increase classification accuracy under low signal-to-noise conditions [52,53]. Hyperspectral imaging (HSI) combined with machine learning (ML), including support vector machine (SVM) approaches, has shown promising results for counting, localizing, and visualizing MPs in different matrices. For the identification of NPs (<1 μm), Raman spectroscopy combined with ML, as well as approaches integrating Gaussian fitting and image reconstruction, may help overcome current optical limitations. As larger reference datasets become available, AI-assisted analytical pipelines may improve reproducibility while reducing operator dependency, thereby facilitating large-scale biomonitoring studies. Given the methodological variability in MP research and the need for harmonized approaches to data-quality screening, generative AI and large language models (LLMs) have also been explored for evaluating study reliability according to QA/QC criteria. Qiu et al. [54] reported strong agreement between LLM-based and human assessments across multiple QA/QC domains, suggesting that these tools may support rapid and consistent methodological quality screening. Nevertheless, such approaches should complement, rather than replace, expert assessment and standardized analytical frameworks.

From a nephrology perspective, future research should prioritize several specific gaps rather than focusing solely on the broader methodological challenges of MP research. First, standardized protocols for the detection and quantification of MPs in urine and kidney tissue are needed to allow meaningful comparisons across studies and institutions. Second, greater attention should be given to smaller particles and nanoplastics, which may represent an important but currently underdetected fraction of renal exposure. Third, longitudinal clinical studies are needed to determine whether MP exposure is associated with validated markers of kidney injury, changes in glomerular and tubular function, or CKD progression, while addressing potential confounding factors and exposure sources. Fourth, patients with CKD and ESKD should be specifically investigated, as these populations may have distinct patterns of exposure and impaired clearance but remain poorly characterized. Finally, computational and AI-assisted approaches should be developed as complementary tools to improve particle characterization, data integration, and methodological quality assessment, while remaining anchored to standardized analytical and clinically validated frameworks. These nephrology-specific priorities are essential to determine whether the detection of MPs in renal and urinary compartments translates into clinically meaningful kidney outcomes.

### 7.2. Experimental Models: Towards More Environmentally Relevant Microplastics

Methodological limitations are not restricted to analytical techniques but also affect experimental study design. Despite the remarkable diversity of environmental MPs, most in vitro studies continue to rely almost exclusively on monodisperse polystyrene (PS) micro- and nanobeads because they are commercially available, easy to characterize, stable in suspension, and frequently fluorescently labeled. While these models have been fundamental for elucidating the basic mechanisms of MPs toxicity, they poorly reproduce real-world human exposure. Moreover, commercial MNP formulations may contain antimicrobial agents and surfactants (such as sodium azide and Tween 20), which may confound toxicity assessments, while fluorescently labelled MNPs may release fluorophores and thereby lead to erroneous estimates of particle uptake and absorption.

Environmental MPs differ substantially from synthetic laboratory beads. Their biological behavior is influenced by multiple physicochemical characteristics, including polymer composition, particle size and morphology, surface charge, environmental ageing, associated additives, and the formation of eco-coronas and protein coronas. These properties determine tissue distribution, cellular uptake, intracellular trafficking, and ultimately biological toxicity. Because plastics may persist for prolonged periods in the environment and in biological tissues, they can progressively release additives and degradation products, including phthalates and other plasticizers [55,56]. Understanding degradation processes and compound leaching from MPs is therefore essential for improving the assessment of environmentally relevant toxicity. Consequently, data generated using pristine PS beads may not accurately reflect the biological effects of environmentally relevant MPs. Moreover, MP concentrations used in in vitro and animal studies often differ by several orders of magnitude from estimated human exposure concentrations (µg/mL or mg/L versus ng/L or µg/L). Such supraphysiological concentrations are frequently used in toxicological studies to compress chronic processes into experimentally tractable time frames. These results should therefore be regarded as mechanistically informative rather than as direct estimates of human risk. Two additional considerations are important. First, the true clearance kinetics of MPs from different tissues and organs in vivo remain poorly defined, and tissue concentrations may differ substantially from circulating concentrations. Second, in vitro, the dose delivered to a cell monolayer may differ from the nominal administered concentration as a function of particle density, size, aggregation, and sedimentation. Nominal exposure concentrations are therefore not directly comparable with measured tissue concentrations, and no dose–response relationship anchored to MNP concentrations in human renal tissue has yet been established.

Future experimental studies should therefore move toward true-to-life exposure models. Rather than relying exclusively on commercially available PS beads, investigators should prioritize polymers that are more frequently detected in human tissues, such as polyethylene (PE), polypropylene (PP), polyethylene terephthalate (PET), polyvinyl chloride (PVC), and polyurethane (PU). Likewise, environmentally aged particles generated through ultraviolet irradiation or mechanical fragmentation more closely reproduce the physicochemical characteristics of MPs encountered in nature, including irregular morphology, heterogeneous size distribution, surface oxidation, and retention of plastic additives. Recent studies suggest that these environmentally realistic particles exhibit different aggregation properties, interactions with biological matrices, and toxicological profiles compared with pristine laboratory particles [57,58].

Overall, advancing MP research will require not only harmonized analytical protocols but also experimental models that more faithfully reproduce human environmental exposure. The integration of standardized laboratory procedures, computational tools, artificial intelligence, and environmentally relevant experimental materials will be essential to generate reproducible data, facilitate comparisons across studies, and ultimately clarify the true impact of MPs on kidney health.

A summary of the current methodological challenges and future research priorities in MP investigation is provided in Table 4.

## 8. Conclusions

Evidence linking MP exposure to human disease has expanded remarkably over the past decade. MPs have now been detected in multiple human tissues and biological fluids, and accumulating experimental and clinical evidence suggests that they may contribute to cardiovascular, reproductive, neurological, gastrointestinal, and renal disorders. Nevertheless, current knowledge remains largely based on experimental models and relatively small observational studies, precluding definitive conclusions regarding causality, clinically relevant exposure thresholds, and long-term health consequences.

Moreover, a structural asymmetry currently limits this research field: mechanistic evidence is largely derived from nanoscale particles at supraphysiological concentrations, whereas human evidence is mainly restricted to MPs that can be detected by currently available analytical techniques. Experimental and human studies therefore often refer to different particle-size ranges, and their results cannot yet be compared directly.

The kidney occupies a unique position in this emerging field. As the principal organ responsible for filtration and excretion, it is continuously exposed to circulating MPs and may represent both a target organ for particle accumulation and a route of elimination. Experimental studies consistently demonstrate that MPs induce oxidative stress, mitochondrial dysfunction, inflammation, apoptosis, ferroptosis, and fibrosis, while emerging human evidence supports their presence in urine and, in some studies, in kidney tissue. Urinary MPs have consequently emerged as one of the most promising non-invasive biomarkers of human exposure, although their clinical significance and relationship with kidney disease remain to be established.

Among the populations potentially at greatest risk, patients with end-stage kidney disease undergoing dialysis deserve particular attention. Impaired renal clearance, lifelong exposure to large volumes of treated water, and continuous contact with polymer-based extracorporeal devices create a unique scenario that has been only minimally investigated. The few available studies are limited to very small cohorts and are particularly affected by the lack of standardized analytical methods. Patients with CKD and ESKD may thus be among the most exposed and yet least studied populations, and closing this gap should become a specific priority for the nephrology research community.

Future progress will depend on four key priorities: the development of large, well-designed epidemiological studies; the harmonization of analytical methodologies and reporting standards; the integration of computational tools and artificial intelligence to improve the accuracy and reproducibility of MPs detection; and the adoption of environmentally relevant *true-to-life* experimental models that better reproduce human exposure. Addressing these challenges will require close collaboration among nephrologists, toxicologists, environmental scientists, analytical chemists, and computational researchers.

Whether microplastics will prove to be clinically relevant nephrotoxins remains uncertain, but answering this question may define one of the next frontiers of nephrology.

## Figures and Tables

**Table 1:** Key first experimental studies reporting evidence of MPs in kidney tissue and urine with reported differences in analytical approaches.

	Massardo 2024 [7]	Pironti 2023 [16]	Horvatits 2022 [35]	Zhang 2025 [17]
Matrix (n)	Kidney (10, healthy pole, tumour nephrectomy); urine (10, healthy donors)	Urine (6, healthy)	Liver 11, kidney 3, spleen 3 (autopsy/explant)	Kidney tumour 31, peritumoral 8 (Py-GC/MS); 2 pairs (LDIR)
Digestion	10% KOH, 60 °C, 5 d	10% KOH, 40 °C, 48 h	KOH 10 M + NaOCl, 40 °C, 72 h; then H_2_O_2_ + acetone	HNO_3_ 68%, 25 °C 46 h + 60 °C 3 h (LDIR); 40 °C 48 h (Py-GC/MS)
Filter cut-off	0.2 µm PTFE	1.2 µm glass fibre	0.45 µm Ag/0.2 µm Al_2_O_3_	13 µm steel mesh (LDIR); glass fibre (Py-GC/MS)
Technique	µRaman 633 nm, whole-filter inspection; HQI + NC vs. Renishaw/SLoPP/IRUG	µRaman (HQI > 80)	Nile red fluorescence + µRaman on 2–6 particles/sample (>70%)	LDIR (>0.65), SEM, Py-GC/MS (11 polymers)
Size window	1–29 µm kidney; 3–13 µm urine	4–15 µm	4–30 µm	20–500 µm (LDIR); mass-based (Py-GC/MS)
Blanks/LOD	12 procedural + reagents + filters; all negative	1 environmental + 1 procedural	7 procedural; LOD ≈ 3 p/g; blank-corrected	3 field blanks; LOD/LOQ in SI; no blank subtraction reported
Output	66 detected/26 identified (6 polymers, 20 pigments)	7 particles (PP, PE, PVC, PVA)	particles/g; kidney < LOD	µg/g (Py-GC/MS); 443–1456 particles/g (LDIR)

**Table 2:** Key experimental studies providing mechanistic insights into micro- and nanoplastic-induced kidney injury.

Study	Experimental Model	Exposure	Main Mechanistic Contribution	Main Kidney Effect
Wang et al., 2021 [19]	Human HK-2 cells; C57BL/6 mice	PS-MPs	Mitochondrial ROS, ER stress, inflammatory signaling, autophagy	Tubular injury and renal dysfunction
Meng et al., 2022 [26]	Chickens	PS-MPs	NF-κB activation; RIP1/RIP3/MLKL signaling	Inflammation and necroptosis
Zou et al., 2022 [22]	Mice	MPs + Cd	Oxidative stress, autophagy, apoptosis	Enhanced nephrotoxicity and fibrosis
Xiong et al., 2023 [21]	Mice; RNA-seq analysis	PS MNPs (80 nm, 0.5 μm, 5 μm)	Size-dependent transcriptional responses	Differential immune and inflammatory responses
Pan et al., 2024 [20]	Renal tubular cell and animal models	PS-MPs	Tubular epithelial senescence; Klotho/Wnt/β-catenin signaling	EMT, TGF-β1 release and renal fibrosis
Xu et al., 2024 [24]	Mice; scRNA-seq analysis	PS-MPs + high-fat diet	Expansion of PF4^+^ macrophages	Pro-fibrotic remodeling of the renal microenvironment
Zhou et al., 2024 [36]	Human iPSC-derived kidney organoids	PS particles	ROS-dependent apoptosis	Impaired nephrogenesis and reduced organoid growth
Zhang et al., 2025 [25]	Mice	PS + BaP co-exposure	Gut–kidney axis; ACSL4/LPCAT3-mediated lipid metabolism	Renal ferroptosis

**Only selected studies providing distinct mechanistic insights are reported. Abbreviations:** ACSL4, acyl-CoA synthetase long-chain family member 4; BaP, benzo[a]pyrene; Cd, cadmium; EMT, epithelial-to-mesenchymal transition; ER, endoplasmic reticulum; HK-2, human kidney-2 proximal tubular epithelial cells; iPSC, induced pluripotent stem cell; LPCAT3, lysophosphatidylcholine acyltransferase 3; MPs, microplastics; NF-κB, nuclear factor kappa-light-chain-enhancer of activated B cells; PF4, platelet factor 4; PS, polystyrene; PS-MPs, polystyrene microplastics; RIP1/3, receptor-interacting protein kinase 1/3; ROS, reactive oxygen species; scRNA-seq, single-cell RNA sequencing; TGF-β1, transforming growth factor beta 1.

**Table 4:** Summary of current methodological challenges and future research priorities in microplastic investigation.

Main Limitation	Future Perspective
Lack of standardized protocols	Harmonized analytical workflows
High contamination risk	Stronger QA/QC procedures and procedural blanks
Heterogeneity of analytical methods	Multimodal integrated approaches
Manual spectral interpretation	AI-assisted/automated spectral analysis
Limited spectral libraries	Shared open-access polymer databases
Predominant use of PS microbeads	Use of environmentally relevant polymers (PE, PVC, PET)
Poor representation of environmental MPs	“True-to-life” fragmented MPs mixtures
Small human cohorts	Large multicenter epidemiological studies
Limited nephrology-focused evidence	Dedicated CKD and dialysis studies
Detection limits above the biologically relevant size range	Nanoscale chemical identification
Supraphysiological exposure concentrations	Dose-anchored designs bracketing measured human tissue levels; reporting of delivered rather than nominal dose

***Abbreviations:*** MPs, microplastics; QA/QC, quality assurance/quality control; PS, polystyrene; PE, polyethylene; PVC, polyvinyl chloride; PET, polyethylene terephthalate; CKD, chronic kidney disease.

## Data Availability

No new data were created or analyzed in this study. Data sharing is not applicable to this article.
